# High-Dose-Rate Interstitial Brachytherapy for Deeply Situated Gynecologic Tumors Guided by Combination of Transrectal and Transabdominal Ultrasonography: A Technical Note

**DOI:** 10.3389/fonc.2021.808721

**Published:** 2022-01-26

**Authors:** Yuri Shimizu, Naoya Murakami, Takahito Chiba, Tomoya Kaneda, Hiroyuki Okamoto, Satoshi Nakamura, Ayaka Takahashi, Tairo Kashihara, Kana Takahashi, Koji Inaba, Kae Okuma, Yuko Nakayama, Jun Itami, Hiroshi Igaki

**Affiliations:** ^1^ Department of Radiation Oncology, National Cancer Center Hospital, Tokyo, Japan; ^2^ Department of Medical Physics, National Cancer Center Hospital, Tokyo, Japan; ^3^ Radiation Therapy Center, Shin-Matsudo Central General Hospital, Matsudo City, Japan

**Keywords:** interstitial brachytherapy (ISBT), gynecologic malignancies, transrectal ultrasonography (TRUS), transabdominal ultrasonography (TUS), image-guided adaptive brachytherapy (IGABT)

## Abstract

**Background and Purpose:**

High-dose-rate interstitial brachytherapy (HDR-ISBT) is recommended to obtain a better local tumor control for uterine cancer patients in specific situations such as bulky lesions, an extension to the lateral parametrium, or tumors with irregular shapes. Our group uses real-time transrectal ultrasonography (TRUS) to guide freehand interstitial needle insertion. Occasionally, target tumors locate deeper beyond the rectum and cannot be visualized by TRUS. CT can guide needles to deeply located tumors, but in such cases, repeated image obtainment is required to achieve ideal needle localization. In this report, we present nine cases of patients who underwent HDR-ISBT for deeply situated tumors guided by a combination of transrectal and transabdominal ultrasonography (TR/TA-US).

**Material and Methods:**

Nine uterine cancer patients whose tumors were located deeper than the reach of TRUS and underwent HDR-ISBT guided by TR/TA-US were presented. All nine cases had no distal organ metastasis and underwent external beam radiation therapy (EBRT) to the pelvic region for 45–50.4 Gy in 25–28 fractions followed by boost HDR-ISBT for deeply situated tumors guided by TR/TA-US.

**Results:**

There were seven cervical cancer and two endometrial cancer patients: six with extensive uterine corpus invasion, one cervical cancer with massive pelvic lymph node metastasis, one cervical cancer with postoperative pelvic recurrence, and one with left ovarian direct tumor invasion. The median follow-up period was 15 months (range 3–28 months). The average clinical target volume at the time of first HDR-ISBT was 131 ml (range 44–335 ml). The linear distance from the vaginal entrance to the deepest part of the tumor at first time brachytherapy of nine cases was 14.0 (9.0–17.0) cm. HDR-ISBT dose fractionation was 24–30 Gy in four or five fractions. Seven out of nine cases had no local recurrence in the follow-up period. One had local in-field recurrence 25 months after HDR-ISBT. Another case with carcinosarcoma could not obtain local control and underwent salvage hysterectomy for a residual uterine tumor 11 months after HDR-ISBT. Four cases had extra-field recurrence in lymph nodes or distant organs.

**Conclusions:**

In brachytherapy for gynecologic malignancies, deeply situated tumors located out of reach of TRUS may obtain favorable local control by HDR-ISBT guided with TR/TA-US.

## Purpose

Brachytherapy plays an important role in cervical cancer for definitive radiation therapy (RT) because it can give a relatively significant dosage while preserving the surrounding organ at risk (OAR) (1, 2). Small tumors that fall within the isodose line produced by traditional intracavitary brachytherapy (ICBT) can be successfully treated (3, 4).

Tumors tend to expand laterally with the cardinal ligament in locally advanced cervical cancer. In specific clinical situations, such as large lesions, extension to the lateral parametrium, or pelvic sidewall, the American Brachytherapy Society (ABS) guidelines recommend high-dose-rate interstitial brachytherapy (HDR-ISBT) for such cervical cancer patients (1). It was also recommended to combine ICBT and a few interstitial needles in this area to help with dosage coverage and tumor control (5–9). The combination of ICBT and ISBT is the so-called intracavitary/interstitial brachytherapy (IC/IS) (10). Usually, tandem and transperineal needle insertion was guided by transrectal ultrasonography (TRUS). However, sometimes tumors located deeper than the rectum need to be visualized by TRUS view. In such cases, there is a risk of accidentally puncturing surrounding normal tissues when inserting a needle blinded beyond the reach of TRUS.

CT can guide needles to deeply located tumors, but in such cases, repeated image obtainment is required to achieve ideal needle localization. In such cases, we used transabdominal ultrasonography (TAUS) to obtain real-time visualization of a tumor and insert a needle to reduce this risk of normal tissue injury and ensure that the needle tip is within the target.

Here, we presented nine cases of patients who underwent HDR-ISBT for deeply situated tumors guided by a combination of transrectal and transabdominal ultrasonography (TR/TA-US).

## Materials and Methods

### Patients

Uterine cancer patients who underwent HDR-ISBT for deeply situated tumors guided by TR/TA-US between January 2019 and November 2020 were included in this report.

### Treatment

The patients were treated with a combination of pelvic external beam RT (EBRT) and brachytherapy. In all cases, EBRT was up to a dose of 50–50.4 Gy at 1.8–2.0 Gy per fraction in 25–28 fractions with 5 fractions per week. The initial 30–41.4 Gy was delivered to the whole pelvis, and then pelvic irradiation was delivered with a 4-cm width of the central shield (CS), reducing OAR exposure. The initiation of CS depended upon tumor shrinkage. After the CS was inserted or EBRT was accomplished, HDR-ISBT was performed in 1–2 sessions/week. In seven of the nine cases, the needle and applicator were removed after irradiation, and applicator insertion was performed at each treatment (multiple implants). In the remaining two cases, more emphasis was placed on the difficulty of the needle insertion technique into the target, and in order to avoid multiple needle insertions, irradiation was performed every day, twice daily irradiation at 6-h intervals without removing the needles, and needle insertion was performed only once throughout the entire period.

Cervical cancer patients were treated with concurrent chemotherapy basically with weekly cisplatin at 40 mg/m^2^, during the EBRT period. Uterine body cancer patients were treated with RT alone.

### High-Dose-Rate Interstitial Brachytherapy Needle Insertion Technique With Transrectal and Transabdominal Ultrasonography

All patients lay in lithotomy position after saddle block anesthesia or local anesthesia and intravenous sedation. For better pelvic visualization of US, Foley catheterization was performed, and 100 ml of normal saline was instilled into the urinary bladder retrogradely to reduce poor visualization due to intestinal gas.

Transperineal freehand interstitial needle insertion was guided by TR/TA-US. The quality of TA-US is dependent on the examiner’s experience. Adequately inflating the bladder with more than 100 ml of saline is essential in order to obtain a favorable image. It is also important to avoid the pubic bone, apply adequate echo jelly, and compress the probe to the abdominal wall. If these points are observed, even residents with limited experience also can insert needles under TA-US guidance.

**Figure 1:**
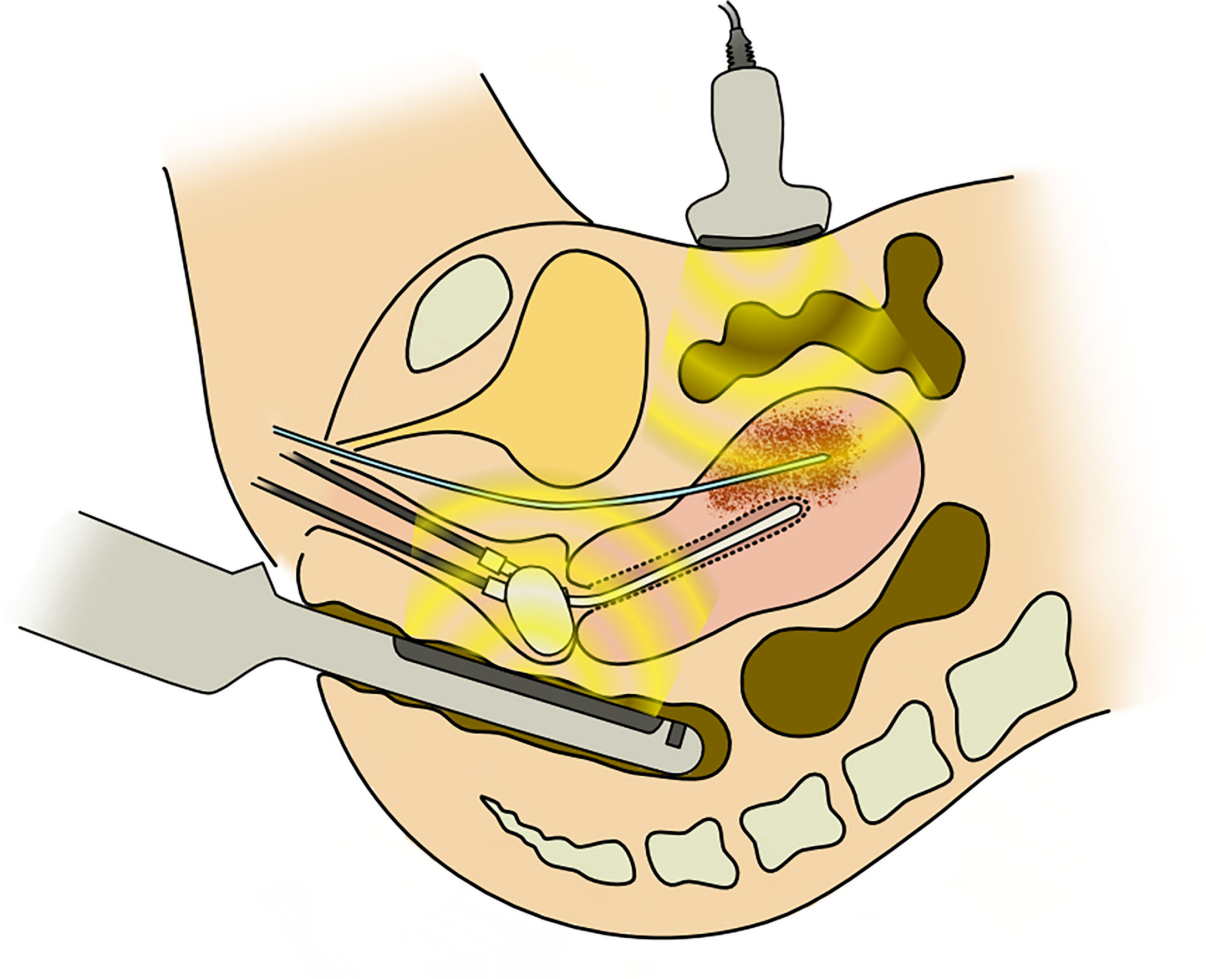
The schema of transabdominal and transrectal ultrasonography-guided interstitial needle insertion.

**Figure 2:**
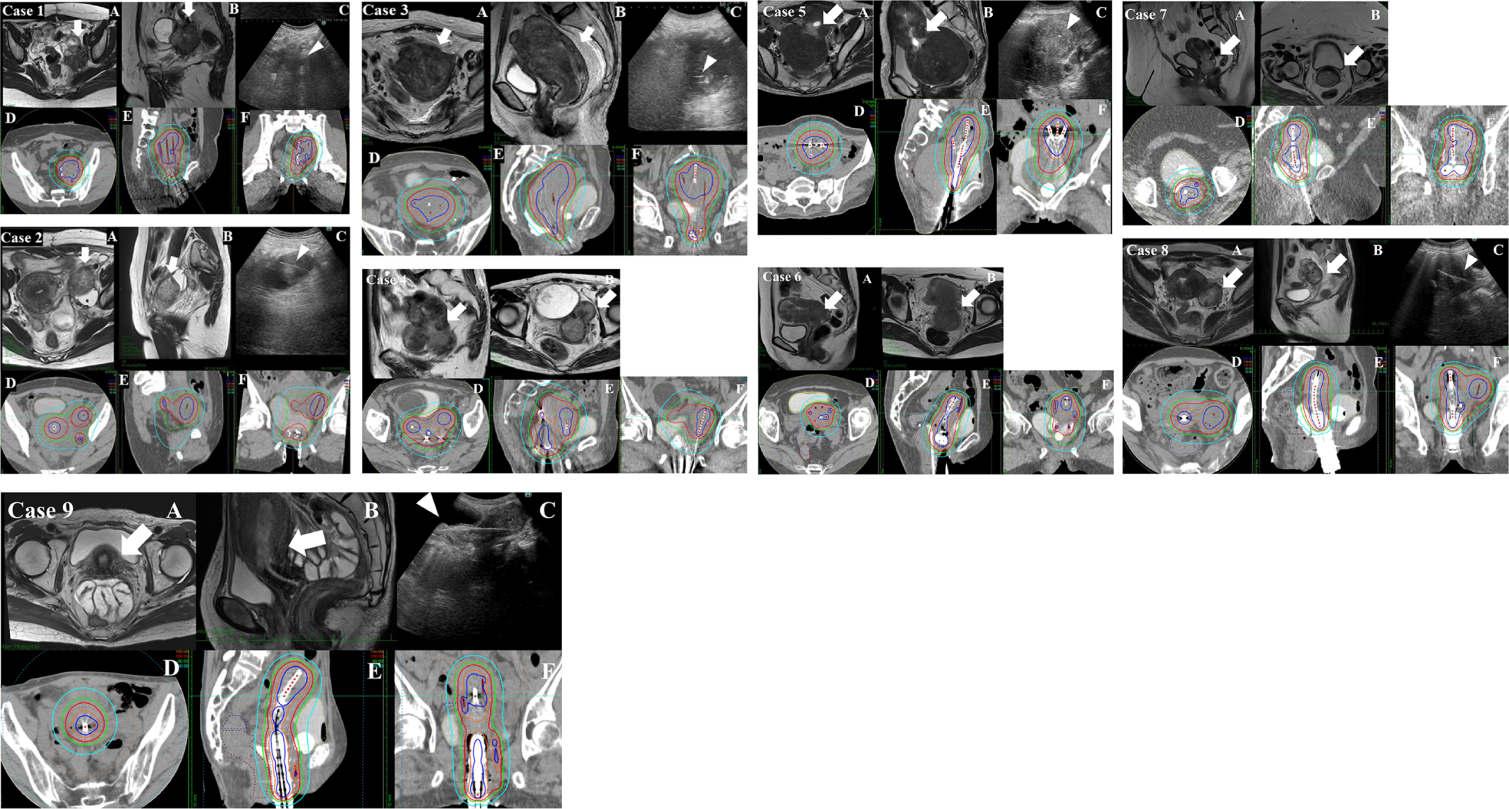
Pre-brachytherapy MRI and brachytherapy dose distribution of cases 1–9. **(A, B)** The transverse and sagittal MRI images at the rectosigmoid junction taken within 1 week before the first brachytherapy. **(C)** The view of transabdominal ultrasonography (TA-US) obtained during the brachytherapy (in cases 4, 6, and 7; TA-US images were unfortunately not saved in the medical record and are lacking). The thick white arrows indicate lymph node metastasis or massive uterine body tumor. The white arrowheads indicate interstitial needles within the tumor. **(D–F)** The brachytherapy dose distribution. The blue, orange, red, green, and sky-blue isodose lines represent 200%, 150%, 100%, 80%, and 50% of the prescription dose (6 Gy), respectively. *Case 1*: A 34-year-old female patient underwent robotic-assisted hysterectomy with right salpingo-oophorectomy and pelvic lymph node dissection in another hospital for cervical squamous cell carcinoma pT1bN1M0 stage 3B according to the 2017 Union for International Cancer Control (UICC) 8th edition classification system. She experienced massive left internal iliac lymph node metastasis. The internal iliac lymph node metastasis was too deep to be visualized by transrectal ultrasonography (TRUS), so TA-US was used for needle insertion guidance. The volumetrically calculated CTV_HR_ and the linear distance from the vaginal entrance to the deepest part of the target tumor at the first brachytherapy were 132 ml and 13.1 cm, respectively. The respective cumulative dose of brachytherapy and external beam radiation therapy (EBRT) was CTV_HR_D_90_ 85 Gy, rectum D_2cc_ 58 Gy, and bladder D_2cc_ 58 Gy. *Case 2*: A 39-year-old female patient with cervical squamous cell carcinoma cT2bN1M1 (para-aortic lymph node metastasis) stage 3C2. Though radical hysterectomy was attempted, she was judged inoperable at the time of laparotomy due to an external iliac node fixed to the external iliac vein. After EBRT, she underwent high-dose-rate interstitial brachytherapy (HDR-ISBT) for the cervical region and also the external iliac node and internal iliac node, which consisted of 24 Gy delivered in four fractional HDR-ISBT doses of 6.0 Gy per fraction in February 2019. The iliac lymph node metastasis was too deep to be visualized by TRUS, and TA-US was used for needle insertion guidance. The volumetrically calculated CTV_HR_ and the linear distance from the vaginal entrance to the deepest part of CTV_HR_ at first time brachytherapy were 86 ml and 13.0 cm, respectively. The respective cumulative dose of brachytherapy and EBRT was CTV_HR_ D_90_ 73 Gy, D_90_ (left external iliac node D_90_ 87 Gy, rectum D_2cc_ 48 Gy, and bladder D_2cc_ 68 Gy. *Case 3*: A 78-year-old female patient with inoperable uterine endometrioid cancer cT3bN2M1 (para-aortic lymph node metastasis) stage 4B. She underwent whole pelvic radiation therapy alone, including a para-aortic node, with a dose was 50.4 Gy/28 fr in November 2019. She underwent HDR-ISBT for huge uterine body cancer, which consisted of 24 Gy delivered in four fractional HDR doses of 6.0 Gy per fraction in December 2019. The uterine body tumor was too large and was too deep to be visualized by TRUS, and TA-US was used for needle insertion guidance. The volumetrically calculated CTV and the linear distance from the vaginal entrance to the deepest part of the tumor at first time brachytherapy were 335 ml and 16.5 cm, respectively. The respective cumulative dose of brachytherapy and EBRT was CTV D_90_ 85 Gy, rectum D_2cc_ 70 Gy, and bladder D_2cc_ 70 Gy. *Case 4*: A 73-year-old female patient with carcinosarcoma of the cervix cT2N1M1a (para-aortic lymph node metastasis) stage 4B. She underwent whole pelvic radiation therapy, including a para-aortic node, with 50.4 Gy/28 fr in July 2019. She had a massive uterine body invasion and needed TA-US because TRUS could not visualize the whole uterine body lesion. After EBRT, she underwent HDR-ISBT for the cervical region, which consisted of 24 Gy delivered in four fractional HDR-ISBT doses of 6.0 Gy per fraction in September 2019. The volumetrically calculated CTV and the linear distance from the vaginal entrance to the deepest part of the tumor at first time brachytherapy were 145 ml and 12.2 cm, respectively. The respective cumulative dose of brachytherapy and EBRT was CTV D_90_ 93 Gy, rectum D_2cc_ 75 Gy, and bladder D_2cc_ 77 Gy. *Case 5*: A 52-year-old female patient with cervical adenocarcinoma cT1b2N1M1a (para-aortic lymph node metastasis) stage 4B. She underwent whole pelvic radiation therapy, including a para-aortic node, with 50.4 Gy/28 fr in January 2020. After EBRT, she underwent HDR-ISBT for the cervical region, which consisted of 22 Gy delivered in five fractional HDR-ISBT in March 2020. She had a massive myoma near the external uterine ostium, which made TRUS unable to see the tumor clearly; therefore, TA-US was required to visualize the lesion. The volumetrically calculated CTV and the linear distance from the vaginal entrance to the deepest part of the tumor at first time brachytherapy were 200 ml and 17 cm, respectively. The respective cumulative dose of brachytherapy and EBRT was CTV D_90_ 80 Gy, rectum D_2cc_ 56 Gy, and bladder D_2cc_ 76 Gy. *Case 6*: A 53-year-old female patient with cervical cancer cT2bN1M1a (para-aortic lymph node metastasis) stage 4B. She underwent whole pelvic radiation therapy, including a para-aortic node, with 50.4 Gy/28 fr in January 2020. After EBRT, she underwent HDR-ISBT for the cervical region, which consisted of 24 Gy delivered in four fractional HDR-ISBT doses of 6.0 Gy per fraction in March 2020. She had a massive uterine body invasion and needed TA-US because TRUS could not visualize the whole uterine body lesion. The volumetrically calculated CTV and the linear distance from the vaginal entrance to the deepest part of the tumor at first time brachytherapy were 130 ml and 12.5 cm, respectively. The respective cumulative dose of brachytherapy and EBRT were CTV D_90_ 85 Gy, rectum D_2cc_ 58 Gy, and bladder D_2cc_ 65 Gy. *Case 7*: A 47-year-old female patient with cervical carcinosarcoma cT1b2N0M0 stage 1B. She was severely obese, which made surgery difficult; therefore, she underwent definitive radiotherapy. She underwent whole pelvic radiation therapy with 50 Gy/25 fr in December 2020. After EBRT, she underwent HDR-ISBT for the cervical region, which consisted of 30 Gy delivered in five fractional HDR-ISBT doses of 6.0 Gy per fraction in December 2020. She had a massive uterine body invasion and needed TA-US because TRUS could not visualize the whole uterine body lesion. The volumetrically calculated CTV and the linear distance from the vaginal entrance to the deepest part of the tumor at first time brachytherapy were 104 ml and 17.4 cm, respectively. The respective cumulative dose of brachytherapy and EBRT were CTV D_90_ 103 Gy, rectum D_2cc_ 85 Gy, and bladder D_2cc_ 71 Gy. *Case 8*: A 55-year-old female patient with cervical adenocarcinoma cT3bN1M1a (para-aortic lymph node metastasis, left ovarian direct tumor invasion) stage 4B. She underwent whole pelvic radiation therapy, including a para-aortic node with 50 Gy/25 fr in November 2020. After EBRT, she underwent HDR-ISBT for the cervical region, which consisted of 24 Gy delivered in four fractional HDR-ISBT doses of 6.0 Gy per fraction in December 2020. She had left ovarian tumor direct invasion and required TA-US because TRUS could not visualize the whole image of the ovarian invasion. The volumetrically calculated CTV and the linear distance from the vaginal entrance to the deepest part of the tumor at first time brachytherapy were 69 ml and 12.2 cm, respectively. The respective cumulative dose of brachytherapy and EBRT were CTV D_90_ 94 Gy, rectum D_2cc_ 70 Gy, and bladder D_2cc_ 75 Gy. *Case 9*: A 58-year-old female patient with inoperable uterine endometrioid cancer cT3bN2M1 (para-aortic lymph node metastasis) stage 4B. She underwent whole pelvic radiation therapy, including a para-aortic node with 45 Gy/25 fr and 14 Gy/7 fr boost irradiation for pelvic lymph node metastasis in December 2020. She underwent HDR-ISBT for huge uterine body cancer, which consisted of 24 Gy delivered in four fractional HDR-ISBT doses of 6.0 Gy per fraction in January 2021. The huge uterine body lesion was too deep to be visualized by TRUS, and TA-US was used for needle insertion guidance. The volumetrically calculated CTV and the linear distance from the vaginal entrance to the deepest part of the tumor at first time brachytherapy were 173 ml and 16.2 cm, respectively. The respective cumulative dose of brachytherapy and EBRT were CTV D_90_ 79 Gy, rectum D_2cc_ 70 Gy, and bladder D_2cc_ 72 Gy.

After needle insertion, ICBT applicators, tandem, and ovoid/cylinder were inserted. **Figure 1** shows the schematic image of the procedure of inserting a needle under TR/TA-US guidance. The geometry of the needles relative to the target tumor, bladder, intestinal canal, and vessels was confirmed visually before inserting needles. In this report, deeply situated tumors are defined as tumors that are located beyond the reach of the rectosigmoid junction and cannot be physically visualized by TRUS (**Figure 2**).

At each brachytherapy session, a CT image of 2-mm slice thickness was taken by a CT simulator (Aquilion™, Toshiba, Tokyo, Japan) situated in the operating room with the patient lying in lithotomy position with the applicators in place. High-risk clinical target volume (CTV_HR_) at HDR-ISBT is delineated on this CT using MRI taken immediately before the initial HDR-ISBT and TRUS as a reference (6, 11, 12). The definition of CTV_HR_ is based on the CT-based contouring guideline proposed by Viswanathan et al. (13). Treatment planning was executed using the planning software Oncentra^®^ (Elekta, Veenendaal, Netherlands). Dose calculation of HDR-ISBT was based on CT. Dose constraints of HDR-ISBT and the goal are to deliver more than 6 Gy to CTV_HR_ D_90_ (dose covering 90% of the CTV_HR_) and total CTV_HR_ D_90_ combining EBRT and brachytherapy to be >85 Gy equivalent dose in 2-Gy fractions (EQD_2_). The rectum and bladder were contoured as OARs. Dose constraints for OARs were determined as follows: bladder D_2cc_ < 90 Gy EQD_2_ and rectum D_2cc_ < 75 Gy EQD_2_.

All brachytherapy was carried out by ^192^Ir remote afterloading system (RALS, Micro Selectron HDR™, Nucletron, Veenendaal, Netherlands).

### Follow-Up

All patients were followed up by CT and/or MRI scans performed 1–3 months after radiotherapy, and physical examination and blood tests were performed regularly every 1–6 months. Adverse events were assessed by Common Terminology Criteria for Adverse Events (CTCAE) version 4.0.

### Statistical Analysis

All analyses were performed using computer software (Excel Statistics for Windows, Microsoft Excel 2012, Social Survey Research Information, Tokyo, Japan).

This study was also approved by the Institutional Review Board of our hospital, the Ethics Committee of National Cancer Center Hospital (approved number 2017-091), according to the ethical standards laid down in the Declaration of Helsinki.

## Results

Nine uterine cancer patients were entered into this report. The patient’s demographics are summarized in **Table 1**. There were seven cervical cancer and two endometrioid cancer patients. Histopathology was three cervical squamous cell cancers, two cervical adenocarcinomas, two cervical sarcomas, and two uterine endometrioid cancers. The median age at treatment was 53 years (range 34–78). All nine cases had no distant metastasis. The breakdown of the deep located target was four cervical cancers with excessive uterine corpus invasion, two uterine endometrioid cancer with inoperable bulky tumors, one cervical cancer with inoperable pelvic lymph node metastasis, one cervical cancer with postoperative pelvic sidewall recurrence, and one cervical cancer with left ovarian direct tumor invasion. Five cases underwent extended whole pelvic irradiation, which included para-aortic lymph node metastasis in the target area. Seven cervical cancer cases were given concurrent platinum-based chemotherapy with EBRT.

**Table 1:** Patient characteristics.

	Age	Diagnosis	Stage	Histology	Concurrent chemotherapy	Target	CTV_HR_ (ml)	Distance between vaginal introitus and deepest part of tumor (cm)	CTV_HR_ D_90_ (Gy, EQD_2_)	Bladder D_2cc_ (Gy, EQD_2_)	Rectum D_2cc_ (Gy, EQD_2_)
Case 1	34	Cervical cancer	pT1b1N1M0	SCC	Cisplatin + fluorouracil	Postop lymph node recurrence	132	13.1	85	58	58
Case 2–1	39	Cervical cancer	cT2bN1M1a	SCC	Cisplatin	Primary	86	9	73	68	48
Case 2–2	–	–	–	–	–	Lymph node metastasis	44	15	87	–	–
Case 2–3	–	–	–	–	–	Lymph node metastasis	2	13	94	–	–
Case 3	78	Uterine body cancer	cT3bN2M0	EC	–	Primary	335	16.5	85	70	70
Case 4	73	Cervical cancer	cT2bN1M1a	Carcinosarcoma	Cisplatin	Primary	145	12.2	93	77	75
Case 5	52	Cervical cancer	cT1b2N1M1a	Adenocarcinoma	Cisplatin	Primary	200	17	80	76	56
Case 6	53	Cervical cancer	cT2bN1M1a	SCC	Cisplatin	Primary	130	12.5	85	65	58
Case 7	47	Cervical cancer	cT1b2N0M0	Carcinosarcoma	Cisplatin	Primary	104	17.4	103	71	85
Case 8	55	Cervical cancer	cT3bN1M1a	Adenocarcinoma	Cisplatin	Primary and left ovarian direct inv.	69	12.2	94	75	70
Case 9	58	Uterine body cancer	cT3bN2M1a	EC	–	Primary	173	16.2	79	72	70

SCC, squamous cell carcinoma; EC, endometrial cancer.

The median follow-up period was 15 months (range 3–28 months). The median volume of CTV_HR_ at first HDR-ISBT was 131 ml (range 44–335 ml). The median linear distance from the vaginal introitus to the deepest part of the tumor at first time brachytherapy was 14.0 cm (9.0–17.0 cm). The prescribed dose of HDR-ISBT was 24–30 Gy in four or five fractions. Median CTV_HR_ D_90_ (EQD_2_) was 85 Gy (73–93 Gy). Median bladder D_2cc_ (EQD_2_) was 71 Gy (58–77 Gy). Median rectum D_2cc_ (EQD_2_) was 70 Gy (48–85 Gy).

An example of an MRI taken within 1 week before the first brachytherapy and TAUS taken during the brachytherapy of three typical cases is demonstrated in **Figure 2A**. The dose distribution of brachytherapy of the same cases is demonstrated in **Figure 2B**. Seven cases had no local recurrence in the follow-up period. One case had in-field recurrence at 25 months after HDR-ISBT in the initially inoperable massive iliac lymph node. Another case with initially inoperable carcinosarcoma could not obtain local control and subsequently underwent salvage simple hysterectomy for a residual uterine tumor at 11 months after HDR-ISBT and finally achieved local control. Four cases had extra-field recurrence in lymph nodes or distant metastases. Acute adverse events were grade 2 fever in two cases, and grade 2 vesical bleeding in one case, and grade 2 vaginal bleeding in one case. Late adverse events were grade 2 vesical bleeding in one case. No organ or vessel injury was noted due to interstitial needle insertion. In one case of acute vaginal hemorrhage, hemoglobin decreased from 10.8 g/dl before treatment to 8.0 g/dl after ISBT, and it improved to 11.0 g/dl after blood transfusion. In another case, bladder hemorrhage was observed in the acute and late stages without hemoglobin decrease. Although the acute bladder hemorrhage was not accompanied by a decrease in hemoglobin, approximately 200 ml of blood loss was observed when the applicators were removed, necessitating blood transfusion. Because the bladder hemorrhage in the later stage was not accompanied by a decrease in hemoglobin, no blood transfusion was administered.

## Discussion

It has been demonstrated that HDR-ISBT should be offered for large tumors with irregular shapes (9, 14–16). Interstitial needles are inserted either by template guided or freehand guided by TRUS or CT. The use of US in application assistance and treatment planning in image-guided brachytherapy (IGBT) had been well acknowledged by Groupe Européen de Curiethérapie of the European Society for Radiotherapy and Oncology (GEC-ESTRO) (17). CT has the advantage of superior visualization compared with US. Kunogi et al. reported that with CT-guided needle insertion, it is even possible to treat iliac lymph nodes (18). However, US has an advantage of real-time image acquisition without exposing radiation to patients and physicians. Real-time image acquisition is a tremendous advantage in inserting interstitial needles into a region where critical structures such as great vessels or bowel exist. TRUS has also long been used for prostate brachytherapy. Therefore, it is familiar with most brachytherapists. If a tumor can be visualized by TRUS, it is possible to treat iliac lymph nodes by TRUS-guided HDR-ISBT (19). However, when a target is located beyond the rectum, it is impossible to visualize the tumor only with TRUS because the TRUS probe cannot be inserted deeper than the rectum. In such cases, switching to TAUS has the advantage of obtaining images for tumors located in the deep pelvis (**Figure 1**). Thibodeau et al. reported the usefulness of TAUS in detecting uterine perforation with tandem and in visualizing interstitial needles in IC/IS (20). With TR/TA-US guidance, it was shown in this report that not only intrauterine needle insertion but also iliac lymph nodes or ovarian direct tumor invasion could be treated with HDR-ISBT.

In this report, the median CTV_HR_ D_90_ for all nine cases was 85 Gy (range 73–103 Gy), which is above the recommended dose by ABS and GEC-ESTRO guidelines (1, 2, 14). Median bladder D_2cc_ (EQD_2_) and median rectum D_2cc_ (EQD_2_) were also below the recommended dose by ABS and GEC-ESTRO guidelines.

Even though patients included in this study had extremely massive tumors or tumors in locations where it is impossible to treat HDR-ISBT with usual methods, seven out of nine patients achieved local control. Moreover, even tumors were located challenging to treat locations; no grade ≥ 3 adverse events were reported related to HDR-ISBT under TR/TA-US guidance. Therefore, it is likely that deeply situated tumors that localized out of the reach of TRUS may obtain favorable dose coverage and have a possibility to achieve a better prognosis by HDR-ISBT guided by TR/TA-US.

In this report, transperineal needle insertion was primarily used. Interstitial transvaginal needles can sometimes be used for uterine body involvement, and the depth of transvaginal needles can be monitored using TA-US. However, due to the space limitation or interference from the other applicators, it is sometimes difficult to change the needle angle freely with the transvaginal approach. Therefore, in our institution, the transperineal approach is generally preferred.

The limitations of this report are that the number of patients included was only nine, the follow-up period was short, and there was no control cohort to compare clinical outcomes of this new method. Therefore, it is difficult to draw firm conclusions from this report. The limiting factor of TA-US is that the deep pelvic lesion is not always well delineated due to bowel gas and other factors such as thick subcutaneous fat tissue, and TA-US cannot be used as real-time needle insertion guidance in such cases. Generally, lymph node or ovarian metastases are boosted by EBRT, and it is unusual for HDR-ISBT to be chosen as a modality to deliver a boost dose. However, we thought that even with the technique of stereotactic body RT, delivering adequate tumoricidal doses to such lesions is difficult. Brachytherapy has the physical advantage of being able to deliver a localized high dose of radiation with rapid fall-off and capable of sparing surrounding adjacent normal tissues, as well as the technical feasibility of inserting interstitial needles with the new technique presented in this article; therefore, we decided to deliver the boost dose with HDR-ISBT for these patients. With this new technique, it is possible to treat challenging diseases that are otherwise impossible to treat with current treatment methods. Further accumulation of patients, experience, and knowledge is needed to implement this novel technique to spread widely.

## Conclusion

In uterine cancer treatment, deeply situated tumors that localized out of the reach of TRUS may obtain favorable dose distribution by HDR-ISBT guided with TR/TA-US.

## Data Availability Statement

The raw data supporting the conclusions of this article will be made available by the authors, without undue reservation.

## Ethics Statement

The studies involving human participants were reviewed and approved by the Ethics Committee of National Cancer Center Hospital (approved number 2017-091). The patients/participants provided their written informed consent to participate in this study. Written informed consent was obtained from the individual(s) for the publication of any potentially identifiable images or data included in this article.

## Author Contributions

TaK, AT, ToK, KT, KI, KO, YN, JI, and HI contributed to patients’ treatment and data collection. TC, HO, and SN contributed to data analysis and figure creation. All the authors contributed to manuscript editing and approved it.

## Funding

We received a grant for the cost of this study from the hospital's collaborative research fund C2019-121.

## Conflict of Interest

JI reports grants from Elekta, during the conduct of the study; personal fees from Heka-Bio; personal fees from Alpha-TAU; grants and others from ITOCHU; and personal fees from Palette Life Science, outside the submitted work. HI reports grants and personal fees from Heka-Bio, grants from CICS, grants from Elekta KK, personal fees from AstraZeneca, personal fees from Itochu, personal fees from HIMEDIC, and personal fees from Varian, outside the submitted work. KI reports personal fees from Boston Scientific Japan, outside the submitted work. TaK reports personal fees from Astra Zeneca, outside the submitted work.

The remaining authors declare that the research was conducted in the absence of any commercial or financial relationships that could be construed as a potential conflict of interest.

## Publisher’s Note

All claims expressed in this article are solely those of the authors and do not necessarily represent those of their affiliated organizations, or those of the publisher, the editors and the reviewers. Any product that may be evaluated in this article, or claim that may be made by its manufacturer, is not guaranteed or endorsed by the publisher.

## References

[1] Viswanathan AN Thomadsen B . American Brachytherapy Society Cervical Cancer Recommendations Committee; American Brachytherapy Society. American Brachytherapy Society Consensus Guidelines for Locally Advanced Carcinoma of the Cervix. Part I: General Principles. Brachytherapy (2012) 11:33–46. doi: 10.1016/j.brachy.2011.07.003 22265436

[2] Viswanathan AN Beriwal S De Los Santos JFD Demanes J Gaffney D Hansen J . American Brachytherapy Society Consensus Guidelines for Locally Advanced Carcinoma of the Cervix. Part II: High-Dose-Rate Brachytherapy. Brachytherapy (2012) 11:47–52. doi: 10.1016/j.brachy.2011.07.002 22265437 PMC3489267

[3] Bethesda M . Dose and Volume Specification for Reporting Intracavitary Therapy in Gynecology. In: ICRU Report 38, vol. 38. Washington: ICRU (1985). p. 1–20.

[4] Tod M Meredith WJ . Treatment of Cancer of the Cervix Uteri, a Revised Manchester Method. Br J Radiol (1953) 26:252–7. doi: 10.1259/0007-1285-26-305-252 13042092

[5] Kirisits C Lang S Dimopoulos J Berger D Georg D Potter R . The Vienna Applicator for Combined Intracavitary and Interstitial Brachytherapy of Cervical Cancer: Design, Application, Treatment Planning and Dosimetric Results. Int J Radiat Oncol Biol Phys (2006) 65:624–30. doi: 10.1016/j.ijrobp.2006.01.036 16690444

[6] Jurgenliemk-Schulz IM Tersteeg RJ Roesink JM Bijmolt S Nomden CN Moerland MA . MRI-Guided Treatment-Planning Optimization in Intracavitary or Combined Intracavitary/Interstitial PDR Brachytherapy Using Tandem Ovoid Applicators in Locally Advanced Cervical Cancer. Radiother Oncol (2009) 2:322–30. doi: 10.1016/j.radonc.2009.08.014 19748695

[7] Dimopoulos JCA Kirisits C Petric P Georg P Lang S Berger D . The Vienna Applicator for Combined Intracavitary and Interstitial Brachytherapy of Cervical Cancer: Aspects of Clinical Feasibility and Preliminary Results. Int J Radiat Oncol Biol Phys (2006) 66:83–90. doi: 10.1016/j.ijrobp.2006.04.041 16839702

[8] Nomden CN DeLeeuw A Moerland MA Roesink JM Tersteeg RJ Jürgenliemk-Schulz IM . Clinical Use of the Utrecht Applicator for Combined Intracavitary/Interstitial Brachytherapy Treatment in Locally Advanced Cervical Cancer. Int J Raiat Oncol Biol Phys (2012) 82:1424–30. doi: 10.1016/j.ijrobp.2011.04.044 21669505

[9] Murakami N Kobayashi K Kato T Nakamura S Wakita A Okamoto H . The Role of Interstitial Brachytherapy in the Management of Primary Radiation Therapy for Uterine Cervical Cancer. J Contemp Brachyther (2016) 8(5):391–8. doi: 10.5114/jcb.2016.62938 PMC5116446 27895680

[10] Wakatsuki M Ohno T Yoshida D Noda SE Saitoh J Shibuya K . Intracavitary Combined With CT-Guided Interstitial Brachytherapy for Locally Advanced Uterine. Cervical Cancer: Introduction of the Technique and a Case Presentation. J Radiat Res (2011) 52(1):54–8. doi: 10.1269/jrr.10091 21293072

[11] International Commission on Radiation Units and Measurements (ICRU) . Dose and. Volume Specification for Reporting Intracavitary Therapy in Gynecology. In: Icru Report 38. Bethesda, MD: ICRU (1985).

[12] Haie-Meder C Pötter R Van Limbergen E Briot E De Brabandere M Dimopoulos J . Gynaecological (GYN) GEC-ESTRO Working Group. Recommendations. From Gynaecological (GYN) GEC-ESTRO Working Group (I): Concepts and Terms in 3D Image-Based 3D Treatment Planning in Cervix Cancer Brachytherapy With Emphasis on MRI Assessment of GTV and CTV. Radiother Oncol (2005) 74(3):235–45. doi: 10.1016/j.radonc.2004.12.015 15763303

[13] Viswanathan AN Dimopoulos J Kirisits C Berger D Pötter R . Computed. Tomography Versus Magnetic Resonance Imaging-Based Contouring in Cervical Cancer Brachytherapy: Results of a Prospective Trial and Preliminary Guidelines for Standardized Contours. Int J Radiat Oncol Biol Phys (2007) 68(2):491–8. doi: 10.1016/j.ijrobp.2006.12.021 17331668

[14] Martinez A Cox RS Edmundson GK . A Multiple-Site Perineal Applicator (MUPIT) for Treatment of Prostatic, Anorectal, and Gynecologic Malignancies. Int J Radiat Oncol Biol Phys (1984) 10(2):297–305. doi: 10.1016/0360-3016(84)90016-6 6706724

[15] Syed AM Puthawala AA Abdelaziz NN El-Naggar M Disaia P Berman M . Long-Term Results of Low-Dose-Rate Interstitial-Intracavitary Brachytherapy in the Treatment of Carcinoma of the Cervix. Int J Radiat Oncol Biol Phys (2002) 54(1):67–78. doi: 10.1016/s0360-3016(02)02900-0 12182976

[16] Nag S Martínez-Monge R Selman AE Copeland LJ . Interstitial Brachytherapy in. The Management of Primary Carcinoma of the Cervix and Vagina. Gynecol Oncol (1998) 70(1):27–32. doi: 10.1006/gyno.1998.5032 9698469

[17] Siebert FA Kirisits C Hellebust TP Baltas D Verhaegen F Camps S . GEC-ESTRO/ACROP Recommendations for Quality Assurance of Ultrasound Imaging in Brachytherapy. Radiother Oncol (2020) 148:51–6. doi: 10.1016/j.radonc.2020.02.024 32335363

[18] Kunogi H Hsu IC Yamaguchi N Kusunoki S Nakagawa K Sugimori Y . Ct- Guided Pelvic Lymph Nodal Brachytherapy. Front Oncol (2021) 19:532555. doi: 10.3389/fonc.2020.532555 PMC7933543 33680907

[19] Yoshida K Ueda M Yamazaki H Takenaka T Yoshida M Miyake S . Interstitial Brachytherapy Using Virtual Planning and Doppler Transrectal Ultrasonography Guidance for Internal Iliac Lymph Node Metastasis. J Radiat Res (2012) 53(1):154–8. doi: 10.1269/jrr.11142 22240939

[20] Thibodeau R Simone BA Tanny S Hahn SS Aridgides PDJ . Advantages of Real-Time Transabdominal Ultrasound Guidance in Combined Interstitial/Intracavitary Cervical Brachytherapy: A Case-Based Review. J Contemp Brachyther (2021) 13(2):211–20. doi: 10.5114/jcb.2021.105290 PMC8060964 33897796

